# Factors Influencing Quality of Life of Obese Students in Hangzhou, China

**DOI:** 10.1371/journal.pone.0121144

**Published:** 2015-03-23

**Authors:** Ying-Ping Chen, Hong-Mei Wang, Todd C. Edwards, Ting Wang, Xiao-Ying Jiang, Yi-Ran Lv, Donald L. Patrick

**Affiliations:** 1 Institute of Social Medicine and Family Medicine, School of Medicine, Zhejiang University, Hangzhou, Zhejiang, People's Republic of China; 2 Department of Health Services, University of Washington, Seattle, Washington, United States of America; TNO, NETHERLANDS

## Abstract

**Objective:**

To evaluate the quality of life (QOL) of overweight and obese middle or high school students and identify relevant factors influencing their QOL scores.

**Methods:**

716 students were recruited from 6 middle or high schools in Hangzhou, China. The Chinese version of the Youth Quality of Life Instrument–Weight Module (YQOL-W) was self administered. The YQOL-W scores were compared among different BMI groups, gender, educational status, annual household income, parental education and recruitment community using *t* test or one-way analysis of variance. The independent association of these variables with QOL among overweight and obese students was examined using multivariable linear regression modeling.

**Results:**

Overweight and obese students reported lower total scores, self, social and environment scores than their normal weight peers (all *P*<0.001). The QOL of overweight and obese middle and high school students was associated with BMI value, gender, educational status, parental education, and recruitment community. Girls had lower total scores, self, social and environment domain scores than boys (all *P*<0.001); high school students had lower total and three domain scores than middle school students (all *P*<0.05). Students whose fathers had higher education reported higher total scores, self and social scores than students with less educated fathers (all *P*<0.05). Students whose mothers had higher education reported higher environment scores than students with less educated mothers (*P* = 0.01). Students from migrant communities reported significantly lower total scores, self and social scores than those from rural communities (all *P*<0.05), but comparable scores with those from urban communities (*P*>0.05). Students from migrant communities reported comparable environment scores with those from rural and urban communities (*P*>0.05).

**Conclusions:**

Overweight and obesity have negative effects on students’ quality of life. Therefore weight specific QOL could be included in weight reduction interventions as a relevant outcome.

## Introduction

Childhood obesity has become a threat to public health in many countries. In the World Health Organization African Region alone, the number of overweight or obese children increased from 4 million in 1990 to 9 million in 2013 [1]. In the United States, 16.9% of children and adolescents 2–19 years of age were obese in 2011–2012 [2]. In South Korea, 10.8% of the child population was obese in 2008, a 2-fold increase from a decade ago [3].

The dramatic worldwide trend towards increased childhood obesity appears to include China. According to data from the National Survey on Students’ Physical Fitness and Health in 2010, the prevalence of obesity in China was 13.33, 5.64, 7.83, 3.78 percent for urban boys, urban girls, rural boys and rural girls respectively, which was 1.94, 0.63, 2.76, 1.15 percent higher than the prevalence in 2005 [4]. Childhood obesity has serious health consequences, including increased risk of heart disease, type 2 diabetes, fatty liver and other obesity related complications that affect virtually every organ [5]. Psychosocial impairments appear to be more prevalent at younger ages however, for example, poor self-esteem and behavioral problems seem to be more common in obese children and adolescents [6, 7]. As obesity rates increase, finding out what youth’s perceptions are regarding their weight is an important step for intervention planning and testing the effectiveness of these interventions. QOL is a broad and global concept affected in complex ways by the person’s physical health, psychological state, level of independence, social relationships, and the person’s relationships to salient features of the environment, such as, living in a safe neighborhood, or availability of parks or opportunities for recreation [8]. A recent review found that obesity is associated with lower QOL in children and adolescents across multiple domains including overall health-related quality of life, social functioning, physical functioning and psychological well-being [9]. Overweight and obese youths are at a risk of developing health-compromising behaviors that may compound medical and social problems associated with excess weight [10]. In China, youth quality of life research is still emerging. A recent review found that among the 238 studies published on youth’s QOL from 1999 to 2008, only 5 of them were related to obesity and few existing studies used established weight-specific QOL instruments for youth [11].

The purpose of this study was to evaluate QOL in a sample of middle and high school students using the Chinese version of Youth Quality of Life Instrument-Weight Module and further identify factors influencing overweight or obese students’ QOL.

## Methods

### Ethics statement

This study was approved by Zhejiang University School of Medicine Ethics Committee. The study’s purpose and all the procedures involved were explained in a youth friendly and understandable way to all potential participants. All participants and their parents (or guardians) provided written informed consent before participation. All data were analyzed without personal identifiers.

### Sample and survey administration

A cross-sectional study was conducted among Chinese students in Hangzhou, Zhejiang Province, from October to December, 2012. Participants were recruited from three middle schools and three high schools in different communities (urban, migrant and rural community respectively). In order to ensure breadth and representativeness, the sample was stratified such that approximately equal numbers of participants were recruited with respect to gender (male, female), age (grades 7–9 and 10–12), recruitment community (urban, migrant, rural) and weight status (normal, overweight, obese) according to body mass index (BMI; kg/m^2^) classification criteria for Chinese youth [12]. In each recruitment cell, potential participants were randomly chosen by simple random sampling based on their latest anthropometric examination and invited by the study coordinator to participate. Teachers handed out leaflets including written informed consent sheets (to be signed by parents/guardians and youth), as well as self screening sheets (by parents/guardians) to those who had verbal consent. They were screened following the exclusion criteria: 1) pregnant or nursing; 2) currently receiving any psychotropic medication; 3) disease history of anorexia nervosa, bulimia, major depression, panic disorders, psychosis, bi-polar disorders; 4) with a life-threatening illness; 5) co-morbid physical disabilities, long-term health problems, or mental health disorders they rated as having a greater impact on their life than their weight. Parents/guardians self-reported basic family information such as parental education and annual household income. All 725 selected students verbally consented and a total of 716 (98.8%) eligible students participated in the study. They were asked to self-administer the Chinese Version of the Youth Quality of Life-Weight Module and have their height and weight measured with metal stadiometer and digital weighing scale. Each measurement was repeated twice (independently by two research assistants), recorded and averaged. If there was greater than 1cm or 1.0kg difference between the two first measurements, a third measurement was taken and the outlier was discarded from an average of the two closest measurements. Students were required to remove shoes, hats or hair ornaments, and heavy clothing that may have interfered with obtaining an accurate measurement. Finally, each student received a $4 gift for their participation.

### Instrument

The Youth Quality of Life Instrument-Weight Module (YQQL-W) was developed by the Seattle Quality of Life Group (SeaQoL), University of Washington as a self-reported instrument to assess weight-specific QOL of children and adolescents 11–18 years of age [13]. The Chinese Version of the YQOL-W was culturally adapted and validated by the Institute of Social Medicine and Family Medicine, Zhejiang University in cooperation with SeaQoL [14]. This 23-item instrument consists of three domains: Self, Social and Environment. The Self domain captures the youth’s feelings about himself or herself, including self-esteem and self-image. The Social domain pertains to youth’s relationships with others including family, friends and peers. Finally, the Environment domain pertains to opportunities and obstacles in a youth’s social and cultural milieu specific to weight status [13, 14]. Each item has eleven response options with adjectival anchors ranging from “0” (Not at all) to “10” (Very Much). Items are then transformed to a 0–100 scale, with higher scores indicating a better quality of life.

### Statistical analysis

Descriptive statistics were calculated to report demographic characteristics of the participants. Continuous variables were presented as means and standard deviations. Categorical variables were presented as observed frequencies and proportions. The YQOL-W scores were compared among different BMI groups, gender, educational status, annual household income, parental education and recruitment community using *t* test or one-way analysis of variance (ANOVA). Given all correlates concerning demographic and social economic status (SES) information, we further examined the independent associations of all these variables mentioned above with the YQOL-W total and subscale scores among overweight and obese students using multivariable linear regression modeling (stepwise regression). All analyses were conducted using SPSS 20.0, results were considered significant at the *P*<0.05 level.

## Results

Among the 716 participants enrolled, 22 with missing data in any YQOL-W item were deleted. The Pearson chi-square test suggested non-significant differences in demographic variables between participants with and without missing data in the YQOL-W (all *P*>0.05). Complete data were available for 694 participants (95.7% of those invited) and analyzed for the present study. 50.3% of participants were middle school students, 49.0% were boys (Table 1). The mean age of the middle school students was 13.4 (SD: 1.01), and the mean age of the high school students was 16.1 (SD: 0.97).

**Table 1:** Demographic Characteristics of the Sample (n = 694^a^).

	n	%
**Educational status**		
Middle school	349	50.3
High school	345	49.7
**Gender**		
Male	340	49.0
Female	354	51.0
**Father’s education**		
Middle school or less	336	48.4
High school or vocational training	222	32.0
Some college	56	8.1
College or higher	64	9.2
**Mother’s education**		
Middle school or less	413	59.5
High school or vocational training	186	26.8
Some college	55	7.9
College or higher	36	5.2
**Annual household income**		
＜60,000 Yuan	274	39.5
≥60,000 Yuan	372	53.6
**Weight status**		
Normal	218	31.4
Overweight	287	41.4
Obese	189	27.2
**Recruitment community**		
Urban community	223	32.1
Rural community	235	33.9
Migrant community	236	34.0

^a^ Sample sizes may not sum to n = 694 due to missing values

Table 2 presented the YQOL-W scores of normal weight, overweight and obese students. One-Way ANOVA revealed significant differences in total and subscale scores across different weight status groups. Pairwise comparisons between weight categories showed that overweight students reported significantly lower total and subscale scores than their normal weight peers, and obese students had the lowest scores compared with normal weight or overweight students (all *P*<0.001).

**Table 2:** YQOL-W Scores for Normal Weight, Overweight and Obese Students (n = 694, $\bar{X}$±SD).

Weight status	n	Self domain	Social domain	Environment domain	Total score
Normal	218	87.59±17.41	93.99±10.20	90.48±15.62	91.43±11.94
Overweight	287	76.26±23.41	85.14±18.94	75.94±24.61	80.84±19.60
Obesity	189	72.07±23.19	80.23±19.14	63.90±26.21	74.90±19.07
F		29.074	35.939	70.325	48.038
*P*		<0.001	<0.001	<0.001	<0.001

Results of the univariate analysis of YQOL-W total and subscale scores of overweight and obese students showed that girls reported significantly lower scores than boys (all *P*<0.001), and high school students reported significantly lower scores than middle school students (all *P*<0.01). Differences among groups with different annual household income, recruitment community or parental education were not significant (Table 3).

**Table 3:** Univariate Analysis of YQOL-W Scores of Overweight and Obese Students (n = 476).

Variables	n	Self domain		Social domain		Environment domain		Total score	
		$\bar{X}$±SD	t(*P*)/F(*P*)	$\bar{X}$±SD	t(*P*)/F(*P*)	$\bar{X}$±SD	t(*P*)/F(*P*)	$\bar{X}$±SD	t(*P*)/F(*P*)
**Gender**									
Male	237	83.67±20.19	9.133	88.34±16.10	6.064	77.35±25.12	5.339	85.01±16.94	7.671
Female	239	65.60±22.90	(<0.001)	78.09±20.56	(<0.001)	65.02±25.27	(<0.001)	72.01±19.92	(<0.001)
**Educational status**									
Middle School	247	79.65±21.63	5.020	85.39±18.54	2.612	74.91±24.56	3.312	81.82±18.73	3.916
High School	229	69.14±24.03	(<0.001)	80.83±19.56	(0.009)	67.12±26.76	(0.001)	74.89±19.89	(<0.001)
**Annual household income**									
<60,000 Yuan	184	74.90±22.07	0.525	82.79±18.62	-0.211	69.58±25.80	-0.796	78.10±18.77	-0.101
≥60,000 Yuan	257	73.74±24.40	(0.600)	83.18±19.76	(0.833)	71.59±26.30	(0.426)	78.29±20.26	(0.920)
**Recruitment community**									
Urban community	152	74.73±22.18	1.774	82.49±19.50	2.789	70.74±26.00	0.042	78.08±19.25	1.695
Rural community	166	76.92±24.01	(0.171)	85.90±17.08	(0.063)	71.13±25.91	(0.958)	80.60±18.56	(0.185)
Migrant community	158	72.03±23.75		81.03±19.15		71.60±25.99		76.65±20.83	
**Father's education**									
Middle school or less	237	74.07±24.11	1.538	81.36±20.71	1.841	69.82±25.80	1.089	77.14±20.86	1.531
High school or vocational training	148	76.96±21.76	(0.204)	86.02±17.07	(0.139)	74.34±25.65	(0.353)	81.23±18.01	(0.206)
Some college	35	67.80±26.42		83.62±16.38		68.43±25.85		76.16±17.50	
College or higher	44	73.86±22.36		82.35±18.40		70.97±26.57		77.79±18.77	
**Mother's education**									
Middle school or less	287	75.12±23.42	0.268	82.82±19.58	0.162	70.59±26.46	0.163	78.35±20.15	0.038
High school or vocational training	125	74.38±22.80	(0.848)	83.39±19.92	(0.922)	71.64±25.40	(0.921)	78.61±19.48	(0.990)
Some college	40	71.71±27.30		83.95±17.06		72.63±24.90		78.25±18.61	
College or higher	23	73.54±19.89		85.40±13.26		73.48±25.40		79.72±15.82	

A multivariable linear regression model was established in which YQOL-W total score was the dependent variable and BMI, gender, own educational status, father’s education and recruitment community were independent variables. Students with higher BMI, girls, and high school students reported significant lower YQOL-W total score, while higher father’s education and rural community residence were associated with a higher YQOL-W total score (Table 4). The multivariable linear regression models for the self and social domain scores showed comparable results to the model for the YQOL-W total score (Tables 5 and 6). In the model for the environment domain, students with higher BMI, girls, and high school students reported significant lower environment score as other models showed, while higher mother’s education was associated with a higher environment score, and recruitment community was not associated with the environment score (Table 7).

**Table 4:** Multivariable Linear Regression of YQOL-W Scores of Overweight and Obese Students^a^ (n = 432).

Variables	Unstandardized Coefficients	Standardized Coefficients	t	*P*
(Constant)	145.29		15.672	<0.001
BMI (continuous variable, kg/m^2^)	-1.689	-0.244	-5.428	<0.001
Girl (Boy as reference)	-13.276	-0.339	-7.644	<0.001
High school student (Middle school student as reference)	-6.545	-0.167	-3.473	0.001
Father's education (ordered categorical variable)	2.605	0.132	2.756	0.006
Recruitment community (Migrant community as reference)				
Urban community	3.358	0.080	1.565	0.118
Rural community	5.345	0.131	2.532	0.012

^a^ F = 17.871, R^2^ = 0.201, *P*<0.001

**Table 5:** Multivariable Linear Regression of Self Domain Scores of Overweight and Obese Students^a^ (n = 432).

Variables	Unstandardized Coefficients	Standardized Coefficients	t	*P*
(Constant)	137.549			
BMI (continuous variable, kg/m^2^)	-1.090	-0.131	-2.941	0.003
Girl (Boy as reference)	-17.274	-0.368	-8.354	<0.001
High school student (Middle school student as reference)	-10.505	-0.224	-4.682	<0.001
Father's education (ordered categorical variable)	2.319	0.098	2.060	0.040
Recruitment community (Migrant community as reference)				
Urban community	2.144	0.042	0.822	0.412
Rural community	6.091	0.125	2.460	0.014

^a^ F = 18.978, R^2^ = 0.211, *P*<0.001

**Table 6:** Multivariable Linear Regression of Social Domain Scores of Overweight and Obese Students^a^ (n = 432).

Variables	Unstandardized Coefficients	Standardized Coefficients	t	*P*
(Constant)	138.962			
BMI (continuous variable, kg/m^2^)	-1.542	-0.227	-4.890	<0.001
Girl (Boy as reference)	-10.580	-0.276	-6.014	<0.001
High school student (Middle school student as reference)	-4.620	-0.120	-2.420	0.016
Father's education (ordered categorical variable)	2.549	0.132	2.662	0.008
Recruitment community (Migrant community as reference)				
Urban community	1.915	0.046	0.863	0.389
Rural community	5.665	0.142	2.689	0.007

^a^ F = 12.349, R^2^ = 0.148, *P*<0.001

**Table 7:** Multivariable Linear Regression of Environment Domain Scores of Overweight and Obese Students^a^ (n = 432).

Variables	Unstandardized Coefficients	Standardized Coefficients	t	*P*
(Constant)	177.312			
BMI (continuous variable, kg/m^2^)	-3.174	-0.346	-7.641	<0.001
Girl (Boy as reference)	-14.304	-0.276	-6.167	<0.001
High school student (Middle school student as reference)	-5.322	-0.103	-2.120	0.035
Mother's education (ordered categorical variable)	3.693	0.125	2.577	0.010
Recruitment community (Migrant community as reference)				
Urban community	0.094	0.002	0.032	0.974
Rural community	0.329	0.006	0.119	0.906

^a^ F = 16.547, R^2^ = 0.189, *P*<0.001

## Discussion

We found that BMI, gender, educational status, parental education and recruitment community are significantly associated with weight-specific QOL. Obese high school girls from migrant communities with less educated parents reported lower QOL scores among the overweight and obese students. The YQOL-W is a well-developed QOL instrument and has been adapted for use in other countries [13, 15–17], and the Chinese version has also been shown to have adequate measurement properties [14]. To the best of our knowledge, this is the first study evaluating youth weight-specific QOL with a well-developed weight-specific QOL instrument in China.

The significant inverse relationship between weight status and QOL has been reported previously [7, 18–20]. Despite the identical conclusion in numerous studies that obese children or adolescents have impaired overall QOL compared with normal weight peers, the results varied with subsets of QOL [9]. A recent review reports that physical and social functioning seems to be most affected, with some evidence to support decrements in emotional functioning and minimal evidence of impaired school functioning [9]. These studies applied generic pediatric QOL measures rather than weight-specific measures. Therefore, they are likely less sensitive to QOL decrements experienced as a result of overweight or obesity [9, 15]. Judging from the findings of our study, it appears that increasing weight status negatively impacts overall and all domains of weight specific QOL.

Numerous studies have reported that females have lower QOL scores in one or more domains [21–24]. Our findings also suggest that overweight and obese girls have lower QOL than boys in all domains. It has been hypothesized that gender difference may occur as girls are generally more sensitive and more concerned with their weight status, making them more vulnerable to psychosocial problems and to experiencing greater decrements in self-esteem than boys, especially in peer and school esteem [24–26]. In addition, our findings suggest that high school students experience lower weight-specific QOL than those in middle school. It has been shown that pubertal development with its attendant physical, psychological, and emotional changes generally has a negative effect on QOL, particularly in girls [27, 28]. When adolescents find themselves in puberty, they often encounter problems in coping with their environment [25]. The maladaptive coping follows a U-shaped curve from childhood to late adolescence, and middle adolescence show the highest levels of maladaptive coping strategies such as resignation, self-criticism, avoidance and distraction [29]. Moreover, high school students are undergoing tremendous pressure in preparing for the national higher education entrance examination. A survey of 2,986 high school students in Shanghai found that academic stress was the biggest stressor and the key cause of poor psychological adaptation [30].

Few studies have described the effects of recruitment community and parental education on students’ QOL. In this study, rural-to-urban migrant students had significantly lower scores than rural students, but comparable scores with urban students except that the environment scores concerning difficulties in dressing and exercising showed no difference between the three subgroups. Since the 1980s, millions of migrant workers sought work and business in urban areas far from home and settled with their families in cities [31]. Students living in urban areas have been found to show more concern about their body type, especially girls [32]. Meanwhile, the female population in the 14 to 18 age group in cities from upper and middle social class backgrounds are more affected by the “thin is beautiful” advertisements aimed at them by commercials [33].

Our study indicated that students whose fathers have higher education had higher total scores, self scores and social scores than students with less educated fathers. And students whose mothers have higher education had higher environment scores than those with less educated mothers. Similar results were found in other studies. Al-Akour et al [34] reported that lower educational level and “unemployed” status of fathers were correlated with lower QOL scores in overweight or obese children. Williams et al [35] found that lower educational level of mothers was associated with significantly lower QOL in overweight children. It is possible that well-educated parents are better informed about the negative effect obesity can have on their children [34], thus pay more attention to their children’s weight management such as diet and physical exercises compared with less-educated parents. Besides, in traditional Chinese families, fathers often have higher education and income than mothers, and are the main authority-figure at home. They often play a more important role in affecting children’s self-esteem, self-identity and social development [36, 37], while mothers response more directly to their children's daily needs.

Annual household income was not associated with QOL in this study. Some prior studies evaluated the influence of household income on children’s oral health related QOL, and found a significant association with higher family income predicting better QOL in children [38, 39]. Low household income was related to material deprivation [38] and such children from poor families had limited access to health care and preventive interventions which might lead to a poorer quality of life [39]. However, these studies were aimed at oral health related QOL other than weight specific QOL. Janicke et al. [40] found there was a significant association between family income and parent-reported QOL in overweight youth, with higher income related to better physical functioning. The YQOL-W was self-reported by the youth about how their weight affects their life, which might be different from parental perspectives.

There are several limitations to this study. First, our findings are validated only for middle or high school students of 11–18 years old residing in Hangzhou, China. Therefore, caution is recommended regarding these findings being replicated in different regions. Second, the study had a cross-sectional design; therefore the data cannot be used to infer causation of the observed associations. Third, The YQOL-W concerns weight specific influences on youth’s life. Other important factors (e.g. diet or physical activity, pubertal development, etc) which could affect generic QOL [27, 41] might also have confounding influence on weight specific QOL which needs to be examined in further studies.

## Conclusions

Overweight and obesity have a negative effect on middle or high school students’ quality of life. Obese high school girls from migrant communities with less educated parents reported lower weight specific quality of life among the overweight and obese students. Future intervention might want to focus attention on this subgroup. Weight interventions should not exclusively include BMI as an outcome, but take perceptual measures of weight-specific QOL into account, which may help to understand what overweight and obese adolescents really concern or worry, and thus increase the effectiveness of these weight interventions including adolescents, their family and communities.

## Supporting Information

S1 Dataset Factors Influencing Quality of Life of Obese Students in Hangzhou, China (SAV). (ZIP)
